# Integrated HIV Testing, Malaria, and Diarrhea Prevention Campaign in Kenya: Modeled Health Impact and Cost-Effectiveness

**DOI:** 10.1371/journal.pone.0031316

**Published:** 2012-02-08

**Authors:** James G. Kahn, Nicholas Muraguri, Brian Harris, Eric Lugada, Thomas Clasen, Mark Grabowsky, Jonathan Mermin, Shahnaaz Shariff

**Affiliations:** 1 Philip R. Lee Institute for Health Policy Studies, University of California, San Francisco, California, United States of America; 2 Ministry of Public Health and Sanitation, Government of Kenya, Nairobi, Kenya; 3 CHF International, Nairobi, Kenya; 4 London School of Hygiene & Tropical Medicine, London, United Kingdom; 5 ESP/UN Foundation, Washington, D.C., United States of America; 6 Coordinating Office for Global Health, CDC, Centers for Disease Control and Prevention-Kenya, Nairobi, Kenya; UCL Institute of Child Health, University College London, United Kingdom

## Abstract

**Background:**

Efficiently delivered interventions to reduce HIV, malaria, and diarrhea are essential to accelerating global health efforts. A 2008 community integrated prevention campaign in Western Province, Kenya, reached 47,000 individuals over 7 days, providing HIV testing and counseling, water filters, insecticide-treated bed nets, condoms, and for HIV-infected individuals cotrimoxazole prophylaxis and referral for ongoing care. We modeled the potential cost-effectiveness of a scaled-up integrated prevention campaign.

**Methods:**

We estimated averted deaths and disability-adjusted life years (DALYs) based on published data on baseline mortality and morbidity and on the protective effect of interventions, including antiretroviral therapy. We incorporate a previously estimated scaled-up campaign cost. We used published costs of medical care to estimate savings from averted illness (for all three diseases) and the added costs of initiating treatment earlier in the course of HIV disease.

**Results:**

Per 1000 participants, projected reductions in cases of diarrhea, malaria, and HIV infection avert an estimated 16.3 deaths, 359 DALYs and $85,113 in medical care costs. Earlier care for HIV-infected persons adds an estimated 82 DALYs averted (to a total of 442), at a cost of $37,097 (reducing total averted costs to $48,015). Accounting for the estimated campaign cost of $32,000, the campaign saves an estimated $16,015 per 1000 participants. In multivariate sensitivity analyses, 83% of simulations result in net savings, and 93% in a cost per DALY averted of less than $20.

**Discussion:**

A mass, rapidly implemented campaign for HIV testing, safe water, and malaria control appears economically attractive.

## Introduction

The potential role of cost-effectiveness analysis in global health decision-making is increasingly recognized [1]. Interventions vary substantially in their ability to deliver health value per amount expended. The value of global health spending can be maximized by prioritizing cost-effective interventions [2].

Differences in cost-effectiveness reflect several factors: the prevalence and severity of disease, the protective effect offered by interventions, and — the only factor substantially under operational control — how efficiently services are delivered. Innovations in delivery strategies may offer substantial savings in cost per person served, as well as greater coverage. These strategies may include a community or health facility focus, as well as streamlining of health care processes [3]–[5]. They can include multiple disease interventions delivered simultaneously, offering the potential to share fixed costs (such as reaching into communities) while addressing multiple high disease burdens. However, little attention has been paid to the economics of multi-disease intervention delivery.

In a separate report, we examined the cost of a multi-disease 7-day integrated prevention campaign (IPC) in Western Province, Kenya, that was implemented in 2008 in 30 village centers [6], [7]. The IPC provided HIV testing and counseling, water filters, insecticide-treated bed nets, condoms, and for HIV-infected individuals CD4 count enumeration, 3 months of cotrimoxazole, and referral to care. Ongoing community mobilization, including health education, was conducted during the campaign, as well as in the preceding month. More than 40,000 (80%) of targeted adults were reached [6]. Full details of the campaign have been published previously [6]. We calculated a cost of $42 per person served for this initial implementation. We further estimated a cost of $32 per person for a scaled-up campaign relying fully on Kenyan staff and using a leaner management structure, as shown possible in a subsequent campaign [7]. The unit cost per person for the scaled-up campaign was estimated to be $6.27 for malaria (nets, education, and training), $15.80 ($2.55 per person-year) for diarrhea (filters, education, and training), and $9.92 for HIV (test kits, counseling, condoms, education, and CD4 testing). These estimated costs compare favorably with prior unit costs of bed nets ($6–27) [8]–[10], filters ($3 per person-year) [11], and HIV VCT ($7–101) [5], [12]–[16].

The cost-effectiveness of these interventions, delivered separately, has been assessed – for example, bed nets $14–42 per disability-adjusted life year (DALY) averted [8], [17], filters $142 per DALY averted [11], and HIV testing $13–18 per DALY averted [13]. The cost-effectiveness of these interventions delivered in combination is unknown. However, provision of a multi-disease intervention package including ART, cotrimoxazole prophylaxis, and bed nets has been shown to provide synergistic benefits for AIDS and malaria [18], [19]. To provide further information for policy makers, we estimated the health impact and cost-effectiveness of delivering an integrated, community-level, multi-disease prevention campaign in rural Kenya.

## Methods

### Overview

We estimated the health impact, cost, and cost-effectiveness of the integrated multi-disease prevention campaign using a spreadsheet-based model constructed for this purpose. We relied on a post-campaign survey to confirm high coverage in the district and to estimate the number of individuals directly benefiting from distributed commodities. We derived baseline morbidity and mortality from regional and local epidemiologic data, and estimated the protective effects of interventions from published community trials. We estimated disability-adjusted life years (DALYs) based on reductions in life years lost to mortality and in illness episodes, weighted by published disability levels. We incorporated an estimated campaign cost of $32 per participant based on financial records and adjustments for scale-up (reported separately and summarized here). Finally, we used published data on costs of health care to estimate savings due to averted disease and added costs due to earlier HIV treatment.

### Model structure

The model was constructed for this analysis in a spreadsheet (Excel, Microsoft Corporation, 2002). The model portrays health benefits and averted costs due to averted disease separately for malaria (due to long-lasting impregnated nets (LLIN)), diarrhea (due to filters), and HIV (due to voluntary counseling and testing (VCT) and condoms). Since these conditions are predominantly unrelated (i.e., in different individuals), we assume independence, which is conservative since interdependence would amplify the health impact of interventions. We used the following calculations:
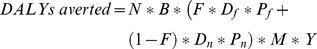
(1)Where:

- N = number who benefit per campaign participant
- B = baseline cases of this disease per year per individual benefiting
- F = proportion of cases that are fatal
- D_f_ = DALYs incurred with each fatality
- P_f_ = protective effect against mortality
- D_n_ = DALYs incurred with each non-fatal case
- P_n_ = protective effect against non-fatal cases
- M = multiplier to capture secondary benefits
- Y = duration of benefit (in years)
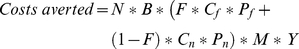
(2)Where:
- C_f_ = costs for health care incurred with each fatality
- C_n_ = costs for health care incurred with each non-fatal case

The model also estimates the health and cost effects of changed level of use of anti-retroviral therapy (ART). The campaign *accelerates* use of ART through earlier detection of infected individuals, with a CD4 count closer to the recommended level for initiation of ART. It may also increase the lifetime use of ART by avoiding deaths prior to HIV diagnosis. This higher ART use extends life (thus averting DALYs), and adds costs. The campaign likely *delays* the use of ART by slowing the decline in CD4 count, through two mechanisms: prevention of malaria episodes, which cause drops in CD4 count; and distribution of cotrimoxazole, which slows CD4 decline. These delays in ART use extend life (averting DALYs) and also avert costs. Finally, we estimate the effect of the net changed time on ART on HIV transmission (which is suppressed by ART use).

Each calculation of the effect on ART use requires several types of inputs: biological factors (e.g., CD4 count at detection of infection, and rates of CD4 decline), intervention effects (e.g., cotrimoxazole effect on rate of CD4 decline), and behaviors (e.g., starting ART if referred). Key input values are presented below. Calculation methods are presented in a technical supplement posted online (Supporting Information S1) and available from the authors.

### Data inputs

Table 1 presents data inputs, with base case values and sources, for the malaria, diarrhea, and HIV prevention analyses. Table 2 presents key data inputs, values, and sources for the analysis of HIV treatment and health status.

**Table 1:** Value of model inputs for prevention, Integrated Prevention Campaign, Western Province, Kenya, 2008.

		Malaria	Diarrhea	HIV		Source(s)		
		LLIN	Filters	VCT	Condoms	LLIN	Filters	VCT/Condoms
**Health inputs**								
N	number who benefit per campaign participant	2.9	3.1	0.95	0.36	Post-campaign survey	Post-campaign survey	Post-campaign survey
B	baseline cases of this disease per year per individual benefiting	0.30	1.75	0.0038	0.009	[18], [20]	[21]–[24]	[25]–[27], Post-campaign survey (see text)
F	proportion of cases that are fatal	0.0033	0.0010	1.0	1.0	[20], [22]	[21]–[24]	Assumption
D_f_	DALYs incurred with each fatal case	30	30	8	8	[24]	[24]	[29]
D_n_	DALYs incurred with each non-fatal case	0.0037	0.0020	n/a	n/a	[24], expert opinion	[24], [28]	N/a
P_f_	protective effect against mortality	0.50	0.63	0.50	0.26	[30], expert opinion	[11]	[31], [32]
P_n_	protective effect against non-fatal cases	0.50	0.63	n/a	n/a	[30]	[11]	N/a
M	multiplier to capture secondary benefits	n/a	n/a	2	2	[34]	N/a	[33] (see text)
Y	duration of benefit (in years)	3	2	1	1	[10], [35]	[36]	[31]
**Cost inputs**								
C_f_	costs for health care incurred with each fatality	$65	$104	$5,092	$5,092	[40], [41]	[42]	[29] (see text)
C_n_	costs for health care incurred with each non-fatal case	$7.80	$7.00	n/a	n/a	[43]	[42]	N/a

**Table 2:** Value of model inputs for treatment and health status in HIV+ individuals, Integrated Prevention Campaign, Western Province, Kenya, 2008.

		Value	Sources
Ae	Seek ART care early	0.60	[26], expert opinion
Ai	Lifetime increase in use of ART due to IPC	0.15	Expert opinion
Ma	Malaria cases averted by LLIN per HIV+ person	0.6	[18], [30]
Ca	CD4 drop averted per malaria event averted (absolute)	40	[19]
Cr	Reduction in CD4 drop with CTX (proportionate)	0.62	[39]
H	HIV infections transmitted per year not on ART	0.05	[27], [31]

#### Health inputs

The number of individuals who benefit per campaign participant for malaria and diarrhea reflects a mean of 2.5 participants per household and 7.7 members per household respectively (both derived from the post-campaign survey). Thus, a mean of 3.1 ( = 7.7/2.5) individuals benefit per campaign participant. This number is lower for malaria (2.9) due to bed nets obtained from non-campaign sources. For HIV, the number benefiting per participant (0.95) is the proportion that were HIV tested, and the number benefiting from condoms (0.36) reflects the percent of participants reporting use of campaign-distributed condoms.

The baseline cases of disease per individual derive from published estimates for sub-Saharan Africa and studies conducted in nearby geographic areas (e.g., Uganda). We estimate 0.3 malaria episodes [18], [20] and 1.75 diarrhea episodes [21]–[24] per person per year. For HIV, estimated annual risk per campaign participant from persons found HIV-positive to others is 0.0038, based on a conservative annual transmission risk of 8% [25] and 4.7% prevalence, from the post-campaign survey and consistent with a national AIDS survey [26]; and to persons found HIV-negative is 0.009, based on HIV incidence imputed from prevalence with assumption of random mixing [27]. Fatality rates for malaria and diarrhea are less than 1% (for all ages, occurring mostly in children) [20]–[24], and for HIV is 100% (over 10 or more years) (assumption).

The DALYs incurred per fatal case of malaria or diarrhea is 28 [24]. For non-fatal cases, the DALY burden is the disability weight (0.192 for malaria; 0.105 for diarrhea) [24] times an assumed duration of disease of 1 week [28]. For HIV the DALY burden of a death is only 8, due to the 12–15 year anticipated survival with antiretroviral therapy [29].

The protective effect of bed nets against malaria mortality is set at 50% (the same as for incidence). This is a conservative estimate based on a 17% reduction in all-cause mortality [30] and malaria representing only 9% of mortality <14 years old [24]. The protective effect of filters on diarrhea is 63% for morbidity [11], and we assume the same for mortality. For HIV, by “death averted” we mean an infection averted. The protection from VCT is conservatively 50% (in positives only) [31] and by condoms is 26% (in negatives only, among the 36% using the condoms, based on the number of condoms provided and 80% protection when a condom is used) [32]. For HIV, each infection averted is assumed to directly avert an average of one additional HIV infection in an epidemic with stable HIV prevalence; this would be a secondary benefit of the HIV interventions in the IPC. To capture this benefit we included a multiplier, which we conservatively set at 2 for HIV, confirmed with analyses using our published epidemic model, which predicts a 2.0 multiplier after 10 years [33]. For malaria and diarrhea, we assumed the interventions used in the IPC (bed nets and filters, respectively) would not have secondary prevention benefits, i.e., each episode averted would not avert any additional episodes. This approach reflects our reliance on community-wide rather than individual effectiveness studies for filters and nets. Further, malaria modeling suggests that current infections averted may not have enduring epidemic benefits [34].

The duration of benefit for bed nets is 3 years [10], [35], and for filters is estimated at 2 years (less than lab data imply) [36]. For HIV risk reduction, one year of benefit is assumed for VCT, reflecting the longest duration of follow-up reported in a recent systematic review [31]. For condoms, we also employed a one-year time frame, using the number of distributed condoms to calculate the incremental probability of protected sex episodes over one year.

For the analysis of *earlier* use of ART, assumptions were as follows (key inputs reported in Table 2). In the campaign, 13 of 88 (14.8%) of a sample of individuals testing positive had a CD4 count less than 250, a commonly used starting level for ART. (We explore the effect of starting ART at CD4<350 through sensitivity analysis). We assumed that 60% of these individuals sought ART care quickly, conservatively one year before they would have otherwise [26]. We estimated the resulting averted DALYs at 0.75, from clinical modeling studies [37], [38]. *Lifetime increased* use of ART due to the IPC (e.g., by avoiding death before HIV diagnosis) is estimated at 15%, based on expert opinion (author JM) considering current and projected lifetime prevalence of ART use. Each additional person on ART averts 7.5 DALYs (discounted, over a lifetime) [29].

Bed nets may *delay* the need for ART by reducing episodes of malaria, which have been associated with an average 40 point decline in CD4 [19]. This results in an approximately one-third year delay to need ART.

Cotrimoxazole also may *delay* the need for ART, based on a study showing a 62% reduction in the rate of decline of CD4 [39]. We apply this reduction after the protection afforded by bed nets, resulting in a further 0.49 year delay in starting ART.

The aggregate effects of the earlier, increased, and delayed ART use described above is 15.1 added years *not* on ART per 1000 campaign participants. This increases HIV transmission by an estimated 0.75 infections per 1000 participants (separate from the HIV prevention effects discussed above). Technical details related to our modeling of the impact of the IPC on HIV treatment are included in a technical supplement (see Supporting Information S1).

#### Cost inputs

We estimated the costs for health care incurred with each malaria and diarrhea fatality based on the direct medical costs of inpatient treatment for each disease, assuming that fatal cases are likely to use inpatient care. The cost of health care (Table 1) is estimated at $65 per fatality for malaria [40], [41] and $104 for diarrhea [42]. For HIV, the costs of health care per fatality are estimated at $5092, based on a 2009 analysis of lifetime costs adjusted for lower annual ART costs ($564) in Zambia in a current analysis [29]. For non-fatal cases, the costs are $7.80 per case for malaria (using a relatively expensive drug, co-artem) [43] and $7 for diarrhea [42], for each assuming outpatient treatment, including the cost of a clinic visit, medications and tests.

The cost of a scaled-up IPC is estimated at $32 per participant [7]. The original campaign cost $42 per participant. A scaled-up campaign will have lower costs through reliance on local rather then foreign managers and a lower manager concentration, as well as returns to scale for the publicity component. These adjustments were initially modeled, and then confirmed in a small subsequent campaign and with price bids for a large campaign [7].

### Sensitivity analyses

We conducted one-way and multivariate sensitivity analyses to assess the importance of uncertainty in input values. To set the uncertainty ranges, we used a 95% confidence interval (CI) when available. For values based directly on empirical data but lacking formal CIs, we used a range of plus or minus one-third of the base case. For values derived indirectly from empirical data or from expert opinion, we used a range of plus or minus one-half. For DALYs due to early death for malaria and diarrhea, we examine down to 25 to reflect potential short-term competing mortality.

The multivariate analysis was a Monte Carlo simulation conducted with Crystal Ball, Decision Engineering © 2000. We used the reported ranges distributed in truncated normal curves. We assumed a 95% correlation of this variable with lifetime cost of treating HIV. Simulation results reflect 100,000 trials.

## Results

### Disease averted

The model estimates that the IPC averts 16.3 deaths: 4.31 from malaria, 6.81 from diarrhea, and 5.22 from HIV. There are an additional 1304 averted episodes of malaria and 6780 of diarrhea.

### DALYs averted

The prevention elements of the campaign avert an estimated 359 DALYs per 1000 participants (Table 3). Most of these benefits (53%) derive from decreased diarrhea, due to the protective effect for relatively frequent disease episodes. Reduced malaria accounts for 35% of averted DALYs, and HIV prevention 12%.

**Table 3:** Results (per 1000 campaign participants), Integrated Prevention Campaign, Western Province, Kenya, 2008.

	Malaria	Diarrhea	HIV		
	LLIN	Filters	VCT	condoms	TOTAL
**Disease averted**					
Deaths	4.31	6.81	5.22		16.3
Episodes	1304	6780	5		8090
**DALYs averted**					
Prevention	125	191	29	13	358.5
Earlier HIV care			82		81.8
TOTAL	**125**	**191**	**125**		**441.8**
**Costs averted (added)**					
Prevention	$10.420	$48,123	$18,169	$8,400	$85,113
Earlier HIV care			($37,097)		($37,097)
TOTAL	**$10.420**	**$48,123**	**($10,538)**		**$48,015**
**Cost-effectiveness**					
Campaign cost (unadjusted)					**$32,000**
Net cost (savings)					**($16,015)**
Cost per DALY averted					**Net savings**

Reduced mortality contributes the vast majority (96%) of DALYs averted through prevention. Though rare, prevented deaths avert 10,000-fold as many DALYs each as do non-fatal disease clinical events.

Earlier HIV care results in a net of 83 averted DALYs. Thus total DALYs averted is estimated at 442 per 1000 campaign participants, 78% of which is from deaths averted.

### Costs averted

The savings due to prevented disease are $85,113 per 1000 participants. The contribution of HIV disease is 31%, much larger than for DALYs due to the high lifetime cost of treatment. Diarrhea and malaria contribute 57% and 12%, respectively.

Earlier HIV care (ART) increases costs by nearly $37,100. Thus, overall savings are $48,015.

### Cost-effectiveness

The estimated campaign cost of $32,000 is less than the savings projected by our model. Thus, in the base case, the campaign is estimated to result in net savings of $16,015 per 1000 participants. With net savings, the incremental cost-effectiveness ratio (ICER) is not reported; an ICER is reported as appropriate in sensitivity analyses. The ICER based on gross costs (unadjusted for offsetting savings) is $72 per DALY averted.

### Sensitivity analyses

We conducted univariate and multivariate sensitivity analyses.

The univariate sensitivity analyses assessed the importance of uncertainty in individual model inputs (Tables 4, 5 and 6). DALYs averted per 1000 participants ranged from 338 to 543. For the 37 inputs assessed, 35 retained net savings for all values; one had a net cost per DALY averted of $0.30, and two had net cost per DALY averted over $1.00: $2.20 and $17.42. The inputs with the largest impact on net costs were the lifetime increase in the use of ART ($32,169 in savings to $143 in added cost); the frequency, magnitude, and duration of benefit for diarrhea; the prevention multiplier and duration of benefit for HIV; and cost per non-fatal diarrhea case.

**Table 4:** One-way sensitivity analyses for health inputs, Integrated Prevention Campaign, Western Province, Kenya, 2008.

			Range used for input		DALYs averted	Net cost (savings)	Cost per DALY averted
			Values	Basis			
**Health inputs: Prevention**			*Base case (BC)*		*442*	*($16,015)*	*Net savings*
N	# who benefit per campaign participant	Malaria (LLIN)	1.9–3.9	±1/3	399–485	($12,436)–($19,622)	Net savings
		Diarrhea (filters)	2.1–4.1	±1/3	381–505	($629)–($31,806)	Net savings
		HIV - VCT	0.9–1.0	±0.05	441–444	($15,057)–($16,969)	Net savings
		HIV - condoms	0. 24–0.48	±1/3	438–446	($13,196)–($18,779)	Net savings
B	baseline cases/year per 1000 persons	Malaria	200–400	±1/3	400–484	($12,540)–($19,486)	Net savings
		Diarrhea	1200–2300	±1/3	382–502	($889)–($31,137)	Net savings
		HIV transmission	2.5–5.1	±1/3	433–452	($9,933)–($22,508)	Net savings
		HIV acquisition	6–12	±1/3	438–447	($13,238)–($18,862)	Net savings
F	proportion of cases that are fatal	Malaria	0.22–0.44%	±1/3	402–482	($15,930)–($16,095)	Net savings
		Diarrhea	0.05–0.15%	±1/3	353–532	($15,683)–($16,343)	Net savings
D_f_	DALYs incurred with each fatal case	Malaria	25 (lower)	see text	429 - BC	= BC	Net savings
		Diarrhea	25 (lower)	see text	423 - BC	= BC	Net savings
		HIV	4–12	±1/2	424–460	= BC	Net savings
D_n_	DALYs incurred with each non-fatal case	Malaria	0.0019–0.0055	±1/2	439–444	= BC	Net savings
		Diarrhea	0.001–0.003	±1/2	435–448	= BC	Net savings
P_f_	protective effect against mortality	Malaria	0.25–0.75	±1/2	382–502	($15,837)–($16,153)	Net savings
		Diarrhea	0.32–0.94	±1/2	354–530	($15,664)–($16,361)	Net savings
		HIV transmission	0.25–0.75	±1/2	428–456	($6,929)–($25,103)	Net savings
		HIV acquisition	0.13–0.39	±1/2	435–449	($11,900)–($20,467)	Net savings
P_n_	protective effect against non-fatal cases	Malaria	0.33–0.67	±1/3	440–443	($12,569)–($19,468)	Net savings
		Diarrhea	0.51–0.72	95% CI	439–444	($7,125)–($22,989)	Net savings
M	multiplier to capture secondary benefits	HIV	1–3	±1/2	421–463	($2,728)–($29,299)	Net savings
Y	duration of benefit (in years)	Malaria	2–4	±1/3	400–484	($12,540)–($19,487)	Net savings
		Diarrhea	1.3–2.7	±1/3	375–509	$824–($32,869)	$2.20 - net savings
		HIV transm.	0.5–1.5	±1/2	421–463	($2,727)–($29,298)	Net savings

Note: BC = base case.

**Table 5:** One-way sensitivity analyses for cost inputs, Integrated Prevention Campaign, Western Province, Kenya.

			Range used for input		DALYs averted	Net cost (savings)	Cost per DALY averted
			Values	Basis			
**Cost inputs**			*Base case (BC)*		*442*	*($16,015)*	*Net savings*
C_f_	costs for health care per fatality	Malaria	$33–$97	±1/2	= BC	($15,875)–($16,151)	Net savings
		Diarrhea	$54–$154	±1/2	= BC	($15,672)–($16,353)	Net savings
		HIV	$2546–$7638	±1/2	= BC	($20,099)–($11,926)	Net savings
C_n_	costs for health care per non-fatal case	Malaria	$3.90–$11.70	±1/3	= BC	($10,943)–($21,083)	Net savings
		Diarrhea	$3.50–$10.50	±1/3	= BC	$7,695–($39,720)	$17.42 - net savings
C_c_	cost of campaign	-	$28,800–$35,200	±1/10	= BC	($19,215)–($12,815)	Net savings

Note: BC = base case.

**Table 6:** One-way sensitivity analyses for inputs on treatment and health status in HIV-positive individuals, Integrated Prevention Campaign, Western Province, Kenya.

			Range used for input		DALYs averted	Net cost (savings)	Cost per DALY averted
			Values	Basis			
**Treatment and health status in HIV+ individuals**			*Base case (BC)*		*442*	*($16,015)*	*Net savings*
Ae	Seek ART care early	HIV	0.3–0.9	±1/2	439–444	($16,658)–($15,368)	Net savings
Ai	Lifetime increase in use of ART due to IPC	HIV	0.075–0.225	±1/2	413–471	($32,169)–$143	Net savings - $0.30
Ma	Malaria cases averted by LLIN per HIV+ person	Malaria-HIV	0.4–0.8	±1/3	441–443	($16,132)–($15,889)	Net savings
Ca	CD4 drop averted per morbid event averted	HIV	13–68	95% CI	440–443	($16,251)–($15,750)	Net savings
Cr	Reduction in CD4 drop with CTX	HIV	0.335–0.905	95% CI	436–447	($16,962)–($15,053)	Net savings
H	HIV infections transmitted per year not on ART	HIV	0.025–0.075	±1/2	431–437	($8,848)–($23,200)	Net savings
Ac	Annual cost of ART	HIV	$282–$846	±1/2	= BC	($17,188)–($14,838)	Net savings

Note: BC = base case.

Uncertainty in baseline cases per 1000 persons (i.e., disease incidence) showed the greatest sensitivity for diarrhea ($889 to $31,137 in net savings), followed by HIV and then malaria (Table 4). The proportion of cases that are fatal (for malaria and diarrhea) had little effect, and DALYs incurred per fatal and non-fatal case also did not affect findings significantly.

Protective effect against mortality strongly affected DALYs but not costs. Even with no mortality benefit for diarrhea, as found in a trial of safe water vessels in the context of weekly clinical monitoring [44], there would be 263 DALYs averted, with net savings of $15,305 (not in table). Protective effect against non-fatal cases has a moderate impact for diarrhea: 51% protection leads to $7,125 net savings.

**Figure 1:**
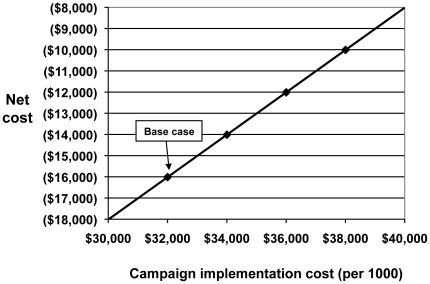
Sensitivity of cost and cost-effectiveness to campaign implementation cost. Integrated Prevention Campaign, Western Province, Kenya, 2008. The base case ($32,000) is cost-saving, and net cost is positive above a campaign cost of $48,000 (not shown; outside of uncertainty range). No cost-effectiveness ratio is calculated, due to net savings.

**Figure 2:**
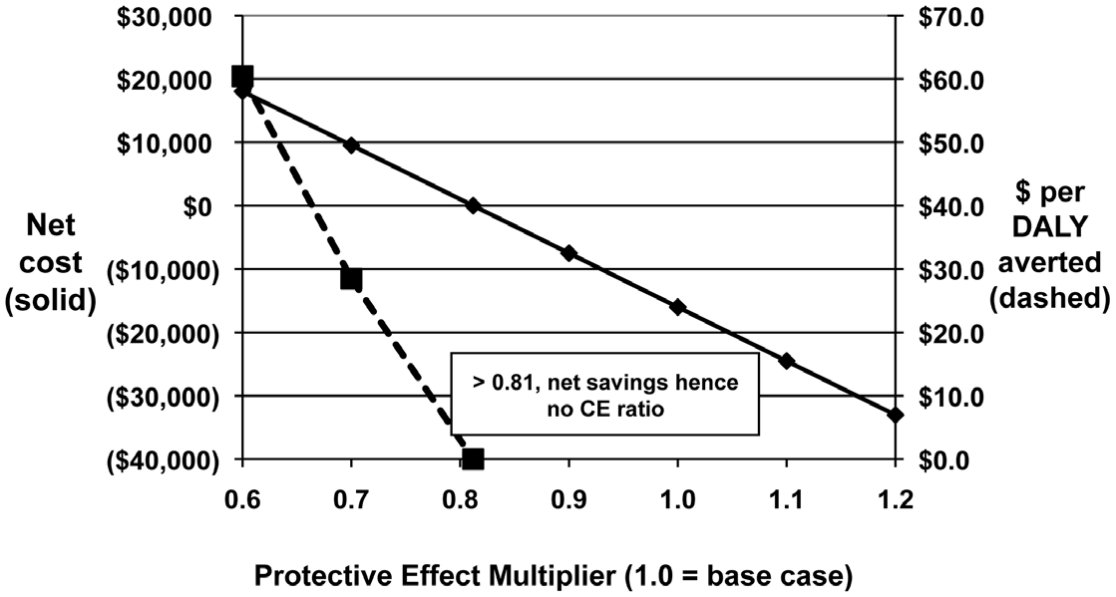
Sensitivity of cost and cost-effectiveness to protective effect (morbidity and mortality). Integrated Prevention Campaign, Western Province, Kenya, 2008. Net cost is positive below 81% of the base case values.

The sensitivity of net cost and cost per DALY averted to campaign implementation cost and protective effect are shown graphically in Figures 1 and 2. The net cost increases as campaign cost rises, but remains negative until the campaign cost reaches $48,000 per 1000 participants (Fig. 1). For the interventions' protective effect, the net cost becomes positive below 0.81 of base case values, and reaches a cost per DALY averted of $60 at 0.6 of base case (Fig. 2).

Due to expanded support for starting ART at CD4<350, we examined the implications of the higher threshold. The effects of earlier ART initiation are modest: DALYs averted increases to 443, and savings drop to $15,539.

Our multivariate analysis (Monte Carlo simulation) suggests that the most likely outcome is substantial health impact with net savings, with only 17% of trials (i.e., calculation iterations) yielding modest costs per DALY averted. The estimated DALYs averted per 1000 participants was mean 442 (standard deviation 78), median 435, 90% confidence interval 327–583, and range 245–641. The mean net savings was $16,102 (median $15,306). The 90% CI was savings of $45,579 to added cost of $10,518; net savings occurred in 83% of trials. The cost per DALY averted was undefined at the mean (due to net savings in most trials), was less than $20 for 93% of trials, and reached a high of $65 per DALY. Graphic results of the multivariate analysis are included in a technical supplement (see Supporting Information S2).

## Discussion

We explored the potential health impact, net cost, and cost-effectiveness of an integrated mass campaign to distribute commodities and services intended to decrease malaria, diarrhea, and HIV. We found, for each 1000 campaign participants, an estimated health benefit of 442 disability-adjusted life years averted, with a net savings of approximately $16,000. The prevention component yielded 81% of the DALYs averted and large net savings ($85,113). Earlier HIV care yielded additional DALYs and also substantial net costs, due to the high cost of ART. Multivariate sensitivity analyses suggest that overall health benefits reside between 327 and 583 DALYs, the campaign is cost-saving for more than four-fifths of simulation trials, and the cost per DALY averted is less than $20 for 93% of trials.

Compared with the cost-effectiveness of individual interventions, these results are generally more favorable. Malaria interventions cost in the range of $2–15 per DALY averted even for the least expensive strategies [1]. Diarrhea prevention has ten- to 100-fold higher cost-effectiveness ratios; filters alone are estimated at $142 per DALY averted [11]. HIV prevention is often cost saving, due to the high cost of care, with savings exceeding costs by 25- to 30-fold [33], [45]. HIV care with ART costs $500–$800 per DALY averted in Africa [29], [46], and CD4 cell and viral load monitoring of ART $174 and over $5000 per DALY averted, respectively [47].

Our analysis had several limitations. As with many cost-effectiveness analyses, health impacts and averted care costs are modeled rather than measured directly for the campaign. However, empirical studies of similar interventions have shown evidence of effectiveness in reducing morbidity and mortality over specified time periods, which we adopted and use as the basis for the modeled prevention benefits from the IPC. By including the best available input values and a diversity of inputs (e.g., protective effects for three diseases) we have mitigated this limitation. Further, robust sensitivity analyses allowed us to assess uncertainty in effectiveness, with favorable findings over the range of values explored.

Second, the campaign cost is based on an economic model for scaling up, and is 25% lower than the cost of the initial campaign implementation. We think that uncertainty in this cost estimate is low, based on confirmatory data from subsequent campaign implementation and planning, and thus has little effect on our findings. However, it will be important to observe actual costs in a scaled up implementation. Repeat campaigns in the same geographical location would yield a lower number of new HIV diagnoses, and depending on timing in relation to commodity life spans could yield lower participation and/or health benefits. Finally, we did not explore savings by linking this campaign to other community campaigns, such as annual mass vitamin A administration [48] or regular indoor residual spraying (IRS) against malaria.

We did not include the effect of earlier ART on tuberculosis (TB). Co-infection of HIV and TB approaches 50% in Kenya [49], and ART may reduce TB acquisition by greater than 60%, depending on when treatment is initiated [50]. This might suggest substantial health gains and economic savings via TB control due to expanded use of ART. In addition, earlier TB diagnosis might lead to easier treatment. However, other factors complicate this picture. Some TB infection that would be detected and treated while under care would never have become clinically significant. Further, ART may induce clinical worsening of TB, due to immune reconstitution (Robin Wood, personal communication). We believe that in the long run, expanded ART will reduce TB transmission and thus prevalence, but in the short run the effects are difficult to anticipate.

This analysis supports a substantial role for integrated multi-disease mass campaigns. Such campaigns are potentially very practical, quickly achieving high coverage of key interventions to reduce the burden of three major diseases, with substantial health benefits, and attractive economics. The campaigns would need to be repeated over time in order to offer ongoing benefits. The optimal timing is unclear, due to the differing duration of campaign interventions: up to ten years for LLIN, three years for water filters, and one year for VCT and condoms. In addition, newly detected HIV cases will drop sharply after the initial implementation, since HIV incidence is much lower than undetected HIV prevalence. Optimal timing would also reflect the local availability of these services through other mechanisms. On balance, we suspect that a three-year cycle would be desirable in most settings. We plan to formally assess this issue in an upcoming analysis.

In conclusion, we propose expanded field implementation of integrated multi-disease mass campaigns, coupled with rigorous evaluation and refinement.

### Disclaimer

The findings and conclusions in this paper are those of the authors and do not necessarily represent the views of the Centers for Disease Control and Prevention.

## Supporting Information

Supporting Information S1 **Technical Supplement 1.** Technical details regarding modeling of the impact of the campaign on HIV treatment.
(DOC)

Supporting Information S2 **Technical Supplement 2.** Technical details regarding Monte Carlo multivariate sensitivity analyses.
(DOC)
