# Supplementary material for: Integrated HIV Testing, Malaria, and Diarrhea Prevention Campaign in Kenya: Modeled Health Impact and Cost-Effectiveness
Source: PLoS One. 2012 Feb 8;7(2):e31316. doi: 10.1371/journal.pone.0031316 (PMC3275624; doi:10.1371/journal.pone.0031316)
Supplement: Supporting Information S1 — Technical Supplement 1. Technical details regarding modeling of the impact of the campaign on HIV treatment. (DOC) [file pone.0031316.s001.doc]

**Technical Supplement 1 for**

Kahn JG et al.

Integrated HIV testing, malaria, and diarrhea prevention campaign in Kenya:
Modeled health impact and cost-effectiveness

In this supplement, we provide exhibits in excess of what could fit in the primary article. This exhibit provides technical details for our modeling of the impact of the campaign on HIV treatment.

Modeling the impact of the IPC on HIV treatment

On the next page is a table that shows the structure and details of our modeling of the effects of the IPC on HIV treatment. We divide the analysis into 5 areas:

1. **Earlier ART due to earlier detection:** We examine the cost and DALY impact of individuals eligible for ART based on CD4 < 250 starting earlier than they would otherwise. Key steps are the proportion eligible (from the campaign), the proportion seeking ART (based mainly on expert opinion by author JM), the mean years earlier that ART is started (also JM), the cost of ART, and the DALYs averted as estimated by ART treatment simulation models.
2. **More ART due to detection:** We model the impact of increasing the number of individuals who *ever* use ART. The lifetime increase in ART use is estimated based on expert opinion (JM), ART lifetime costs, and ART added DALYs.
3. **Malaria prevention – delays need for ART:** This issue affects individuals not yet eligible for ART. Malaria episodes accelerate the decline in CD4, and thus speed the need for ART. We account for the protective effect of LLIN (bed nets) on the number of malaria episodes, and calculate the delay to ART based on the resulting slower CD4 decline. We calculate resulting costs and DALYs averted.
4. **Cotrim (CTX) administration – delays need for ART:** Cotrimoxazole also slows CD4 decline. We an approach similar to that for malaria prevention, except that we apply the CTX reduction only to the CD4 decline that remains after the benefits of LLIN are considered, i.e., we avoid double counting.
5. **HIV transmission due to ART use and delay:** In the final section, we examine the implications of changed ART use on the potential for HIV transmission. We calculate the net added years not on ART (i.e., delay to ART minus increased ART use). This represents time in which HIV can be transmitted, albeit at the lower level expected after testing HIV+ (per Denison 2008).

**Details of modeling of IPC effects on HIV treatment:**

|  | **Parameter** | **Source** | **Value** |
| --- | --- | --- | --- |
|  |  |  |  |
| a | HIV prevalence | community survey | 0.047 |
|  |  |  |  |
| **Earlier ART due to earlier detection** | |  |  |
| b | Eligible for ART, if HIV+ | CD4 testing results from campaign | 0.148 |
| c | Proportion eligible for ART | a * b | 0.007 |
| d | Seek ART care early | extrapolation from Kenya AIDS Indicator Survey / JM | 0.600 |
| e | Number of individuals affected per 1000 IPD participants | c * d * 1000 | 4.2 |
| f | Years earlier on average | estimate since 2 y mortality very high @ CD4 250 / JM | 1 |
| g | Cost per year (ART inclusive, not just drugs) | Zambia Marseille et al pending | $564 |
| h | Added cost per 1000 participants | c * b * f * g * 1000 | **$2,350** |
| i | DALY averted per person starting ART early |  | 0.75 |
| j | DALYs averted per 1000 IPD participants | i * e | 3.1 |
|  |  |  |  |
| **More ART due to detection** | |  |  |
| k | Lifetime increase in use of ART due to IPC | expert opinion / JM | 0.15 |
| l | Number of individuals affected per 1000 IPD participants | k * a * 1000 | 7.1 |
| m | ART cost (lifetime) | , Marseille pending Zambia | $5,092 |
| n | Added cost per 1000 participants | l * m | **$35,899** |
| o | DALY averted due to ART |  | 7.5 |
| p | DALYs averted per 1000 IPD participants | l * o | 52.9 |
|  |  |  |  |
| **Malaria prevention - delays need for ART** | |  |  |
| q | % benefiting (not yet ART eligible) | 1 - b | 0.852 |
| r | Number benefitting | a * q * 1000 | 40 |
| s | Malaria cases averted per HIV+ person per year * duration | case rate * 2 for HIV * protective effect of nets | 0.6 |
| t | CD4 drop averted per morbid event averted |  | 40.0 |
| u | CD4 drop averted, per person benefiting, on average | s * t | 24.0 |
| v | CD4 drop per year | Bendavid personal communication | 70.0 |
| w | Years delay to ART with CarePack (except CTX portion) | u / v | 0.34 |
| x | ART cost (lifetime) | past research / JGK | $5,092 |
| y | ***Savings*** due to delay / discounting, per person | x - x / (1.03^w) | $51 |
| z | ***Savings*** per 1000 IPD participants | r * y | **$2,057** |
| aa | DALY averted per person delaying ART | w | 0.343 |
| bb | DALYs averted per 1000 IPD participants | r * w | 13.7 |
|  |  |  |  |
| **Cotrim (CTX) administration -- delays need for ART** | | |  |
| cc | % benefiting (not yet ART eligible) | 1 - b | 0.852 |
| dd | Number benefitting | a * cc * 1000 | 40 |
| ee | CTX % taking * duration (yrs) | assumption - 60% uptake * 2 years mean use | 1.2 |
| ff | Reduction in CD4 drop with CTX |  | 0.62 |
| gg | Reduction if only CTX, accounting for % taking | ee * ff | 0.744 |
| hh | Percent of CD4 drop already avoided by nets | u / v | 0.34 |
| ii | Reduction if CTX is marginal to filter / bednet effects | gg * (1 - hh) | 0.489 |
| jj | CD4 drop per year | v | 70.0 |
| kk | CD4 drop averted, per person benefiting, on average | ii * jj | 34.2 |
| ll | Years delay to ART with CarePack (CTX portion) | kk / jj | 0.489 |
| mm | ART cost (lifetime) | x | $5,092 |
| nn | ***Savings*** due to delay / discounting, per person | mm - mm / (1.03^ll) | $73 |
| oo | ***Savings*** per 1000 IPD participants | dd * nn | **$2,926** |
| pp | DALY averted per person delaying ART | ll | 0.489 |
| qq | DALYs averted per 1000 IPD participants | dd * pp | 19.6 |
|  |  |  |  |
| **HIV transmission effects due to ART use and delay** | | |  |
| rr | Added years of life | j + p + bb + qq | 89.3 |
| ss | Added years of ART (when otherwise alive not on ART) | j * f / i + l * 2 | 18.3 |
| tt | Added years not on ART (when otherwise alive on ART) | bb + qq | 33.3 |
| uu | **Net** added years not on ART | tt - ss | 15.0 |
| v v | HIV infections transmitted per year not on ART | , calculation | 0.05 |
| ww | Infections transmitted | uu * v v | 0.75 |
| xx | DALYs incurred | ww * 8 | 6.01 |
| yy | Cost | ww * mm | $3,828 |

**References from table:**

1. Walensky RP, Wolf LL, Wood R, Fofana MO, Freedberg KA, et al. (2009) When to start antiretroviral therapy in resource-limited settings. Ann Intern Med 151: 157-166.

2. Bendavid E, Young SD, Katzenstein DA, Bayoumi AM, Sanders GD, et al. (2008) Cost-effectiveness of HIV monitoring strategies in resource-limited settings: a southern African analysis. Arch Intern Med 168: 1910-1918.

3. Marseille E, Kahn JG, Pitter C, Bunnell R, Epalatai W, et al. (2009) The cost effectiveness of home-based provision of antiretroviral therapy in rural Uganda. Appl Health Econ Health Policy 7: 229-243.

4. Mermin J, Lule JR, Ekwaru JP (2006) Association between malaria and CD4 cell count decline among persons with HIV. J Acquir Immune Defic Syndr 41: 129-130.

5. Mermin J, Lule J, Ekwaru JP, Malamba S, Downing R, et al. (2004) Effect of co-trimoxazole prophylaxis on morbidity, mortality, CD4-cell count, and viral load in HIV infection in rural Uganda. Lancet 364: 1428-1434.

6. Granich RM, Gilks CF, Dye C, De Cock KM, Williams BG (2009) Universal voluntary HIV testing with immediate antiretroviral therapy as a strategy for elimination of HIV transmission: a mathematical model. Lancet 373: 48-57.

7. Denison JA, O'Reilly KR, Schmid GP, Kennedy CE, Sweat MD (2008) HIV voluntary counseling and testing and behavioral risk reduction in developing countries: a meta-analysis, 1990--2005. AIDS Behav 12: 363-373.
