# Supplementary material for: Integrated HIV Testing, Malaria, and Diarrhea Prevention Campaign in Kenya: Modeled Health Impact and Cost-Effectiveness
Source: PLoS One. 2012 Feb 8;7(2):e31316. doi: 10.1371/journal.pone.0031316 (PMC3275624; doi:10.1371/journal.pone.0031316)
Supplement: Supporting Information S2 — Technical Supplement 2. Technical details regarding Monte Carlo multivariate sensitivity analyses. (DOC) [file pone.0031316.s002.doc]

**Technical Supplement 2 for**

Kahn JG et al.

Integrated HIV testing, malaria, and diarrhea prevention campaign in Kenya:
Modeled health impact and cost-effectiveness

In this supplement, we provide exhibits in excess of what could fit in the primary article. This exhibit provides technical details for our Monte Carlo multivariate sensitivity analyses.

Monte Carlo multivariate sensitivity analyses – graphics

Here, we present the graphic representations of the Monte Carlo simulation. Each chart shows the frequency of values for one outcome for the 100,000 trials run in the simulation.

The first chart shows the distribution for DALYs. The estimated DALYs averted per 1000 participants was mean 442 (standard deviation 78), median 435, 90% confidence interval 327-583, and range 245-641.

The second chart shows the distribution for net cost. The mean net savings was $16,102 (median $15,306). The 90% CI was savings of $45,579 to added cost of $10,518. As implied in the second cost/DALY chart, net savings occurred in 83% of trials.

The cost per DALY averted was undefined at the mean (due to net savings in most trials), was less than $20 for 93% of trials, and reached a high of $65 per DALY.

83% of trials had a net savings.
